# Antimicrobial peptides: utility players in innate immunity

**DOI:** 10.3389/fimmu.2012.00325

**Published:** 2012-10-25

**Authors:** Mark W. Robinson, Andrew T. Hutchinson, Sheila Donnelly

**Affiliations:** ^1^School of Biological Sciences, Queen's UniversityBelfast, Northern Ireland; ^2^School of Medical and Molecular Biosciences, University of Technology SydneySydney, NSW, Australia; ^3^The ithree Institute, University of Technology SydneySydney, NSW, Australia

Antimicrobial peptides (AMPs) are an ancient group of molecules that are expressed in many species ranging from bacteria to humans. At least 1200 naturally-occurring AMPs have been identified which display considerable diversity in their primary sequences, lengths, structures, and biological activities (Wang et al., 2009). Generally speaking, these peptides have been studied due to their ability to directly kill medically important-microbes. Indeed, the increased demand for novel anti-infective therapies (due to the spread of drug-resistant bacteria) has likely contributed to the expansion of this field of research. However, there has been a definite change of focus in AMP research in light of the recent recognition of the regulatory functions of these molecules in the innate immune system. This has opened up the exciting possibility of developing novel antimicrobial therapies that centre on boosting host immunity rather than direct microbial killing (Zhang and Falla, 2010). In this volume, with emphasis on emerging technologies, mechanism of action studies and roles in disease, we aim to consolidate some of these developments and stimulate discussion on the therapeutic potential of these important molecules.

The first three articles demonstrate how knowledge of AMP structure and amino acid composition is guiding rational peptide design strategies aimed at improving the therapeutic potential of these molecules. Mishra and Wang describe the development of a comprehensive AMP database that is enabling us to link amino acid composition with specific peptide activities (e.g., antibacterial, antifungal, antiviral, antiparasital, insecticidal, spermicidal, anticancer, etc.). The article suggests how this information may be used to develop novel peptides with a desired activity. Next, Scorciapino and Rinaldi discuss how knowledge of the amino acid sequence of naturally-occurring AMPs can be used to dictate the design of antimicrobial peptidomimetics. The authors note that by retaining the biological activity of natural AMPs and improving their pharmacokinetic properties, these novel molecules may allow systemic use of AMPs to treat microbial infections. The third article by Devocelle describes how conjugation of AMPs to targeting moieties can improve delivery to their desired site of action and reduce potential off-target effects. It is clear that the complementary approaches described in these three articles will play an important part in making therapeutic AMPs a reality.

The next two articles describe different, but equally powerful, approaches that may be used to probe the biological functions of AMPs. The development of various “–omics” (genomics, transcriptomics, proteomics, and others) technologies has truly revolutionised biological research. In this regard, Plichta et al. discuss how the integration of various—omics datasets can help us to understand the role of AMPs in varied contexts from resolution of infections, improvement of prognosis for cancer patients to early detection of transplant rejection. The fifth article by Munoz and Read highlights the use of live cell imaging to study AMP function. With emphasis on the various approaches that may be used to label AMPs, this article demonstrates the central role live cell imaging continues to play in the elucidation of AMP function.

The next six articles in this research topic cover recent advances in our understanding of the biological role of AMPs and their mechanism(s) of action. Brender et al. examine the role of cholesterol in dictating the selectivity of AMPs for microbial membranes. Defining the role of cholesterol in AMP-mediated cytotoxicity is important, especially if AMPs are to be used therapeutically. Melo and Castanho discuss the various experimental approaches that may be used to answer this very question. By comparing the use of bacterial membranes with model liposomal membranes, they illustrate the importance of physiological relevance in experimental design. The next two articles in this series illustrate how helminths express AMP homologues that are functionally adapted to either a free-living or parasitic lifestyle. For example, Pujol et al. describe the array of AMPs expressed by the model nematode *Caenorhabditis elelgans* and how they serve to protect the worm against attack by fungi in the free-living environment. Next, Cotton et al. probe the role of AMP homologues that are secreted by medically-important parasitic worms. This interesting family of molecules may hold the key to the evolution of immunomodulatory function in AMPs. In the following two articles, Ulm et al. and Choi and Mookherjee discuss advances in our understanding of the molecular mechanisms by which members of these major AMP families influence immune cell function.

The final two articles in the volume cover the role of AMPs in disease resolution and the outlook for their therapeutic use in humans. The article by David describes how AMPs may fill a gap in our existing therapeutic repertoire. David builds the case for the use of polymyxins to treat Gram-negative sepsis, a condition for which there is currently no single effective therapeutic approach. The article by Berditsch et al. concludes the volume and describes how, perhaps surprisingly, AMPs can stimulate bacterial survival mechanisms leading to persistent infections. This article highlights the importance of understanding how microbial populations respond to exposure to AMPs before they can be used in the clinic.

In summary, by inviting opinion articles from leading AMP researchers, the aim of this volume was to highlight recent advances in our understanding of the roles of these important molecules. We also hope that the articles compiled will stimulate discussion and further research in this area so that the therapeutic potential of AMPs may be realised.
